# Use of Magnetic Resonance Direct Thrombus Imaging for the Diagnostic Management of Suspected Thrombosis in Routine Clinical Practice

**DOI:** 10.1055/a-2341-6349

**Published:** 2024-07-09

**Authors:** Cindy M.M. de Jong, Lisette F. van Dam, Charlotte E.A. Dronkers, Jeroen Eikenboom, Paul L. den Exter, Sophie N.M. ter Haar, Guido R. van Haren, Menno V. Huisman, Thijs E. van Mens, J. Lauran Stöger, Lucia J.M. Kroft, Frederikus A. Klok

**Affiliations:** 1 Department of Medicine—Thrombosis and Hemostasis, Leiden University Medical Center, Leiden, the Netherlands; 2 Department of Emergency Medicine, Franciscus Gasthuis and Vlietland, Rotterdam, the Netherlands; 3 Department of Internal Medicine, Haaglanden Medical Center, Den Haag, the Netherlands; 4 Department of Radiology, Leiden University Medical Center, Leiden, the Netherlands

**Keywords:** venous thrombosis, deep vein thrombosis, magnetic resonance imaging, diagnostic imaging, radiology

## Abstract

**Background** The noninvasive magnetic resonance direct thrombus imaging (MRDTI) technique can be used to diagnose acute deep vein thrombosis (DVT), without the use of intravenous contrast. MRDTI holds the potential to differentiate between acute and chronic DVT and could be helpful when diagnosing thrombosis is challenging.

**Objectives** Our objective was to evaluate the application of MRDTI in clinical practice, including the frequency and indications of MRDTI scans performed in practice-based conditions, results, impact on treatment decisions, and associated patient outcomes.

**Methods** A retrospective study was performed at the Leiden University Medical Center, the Netherlands. MRDTI scans performed since its implementation in patients aged ≥18 years as part of clinical practice for the diagnostic management of suspected thrombosis were evaluated.

**Results** Between October 2015 and September 2023, 36 patients had undergone MRDTI for the diagnostic evaluation of thrombosis. MRDTI application increased since 2019 (five–eight scans per year). The most common indication was to differentiate between acute and chronic thrombosis, mainly for suspected recurrent ipsilateral DVT after inconclusive compression ultrasonography. In over a third of patients, acute thrombosis was confirmed by MRDTI. MRDTI results determined treatment decisions in all except two patients. One patient had symptomatic thrombosis of the lower extremity within 3 months after an MRDTI of the upper extremity without signs of acute thrombosis (1/23; 4.3%, 95% confidence interval: 0.77–21).

**Conclusion** Over the past 4 years, MRDTI has been used increasingly in our hospital. MRDTI results guided treatment decisions, which confirms the clinical impact and feasibility of its application in daily practice.

## Introduction

Magnetic resonance direct thrombus imaging (MRDTI) is a noninvasive magnetic resonance imaging (MRI) technique that can be used to diagnose acute deep vein thrombosis (DVT) by visualizing the metabolism of a fresh thrombus, without involving radiation exposure or intravenous contrast. 1 2 3 The formation of methemoglobin in fresh thrombus causes a shortening of the T1 relaxation time, which produces a high signal from the intravenous thrombus against the suppressed background on a T1-weighted sequence. 1 2 A high signal intensity has been observed to be visible early after clot formation—within 8 hours—, to plateau after approximately 3 weeks, and normalize over a period of 6 months. 2 4 5

As MRDTI imaging has the potential to be used to visualize deep veins inaccessible for compression ultrasonography (CUS) and can be performed in patients with allergies or contraindications for contrast, the technique offers opportunities when diagnosis of thrombosis is challenging. Also, estimating the age of thrombi based on the signal intensity that changes over time holds the potential to differentiate between acute and chronic DVT and to monitor its response to treatment. Therefore, MRDTI could play a role in specific clinical situations such as the differentiation between residual or chronic thrombi and acute thrombosis, suspected thrombosis in the deep veins within the pelvis—whether or not during pregnancy—and the diagnosis of upper extremity DVT. 6 7 8 9 10 The safety of MRDTI as a diagnostic test for excluding acute recurrent ipsilateral DVT of the leg was evaluated in the prospective management Theia study (NCT02262052). 6 MRDTI proved to be a feasible and reproducible diagnostic test and was suggested to be considered in patients with suspected recurrent ipsilateral DVT and a CUS inconclusive for the diagnosis of recurrence. 3 6 MRDTI was also shown to be accurate for the detection of upper extremity DVT. 9

Over the past few years, MRDTI scanning has been performed in routine practice at the Leiden University Medical Center (LUMC). We set out to evaluate the use of MRDTI in our hospital by reviewing indications, test results, impact on treatment decisions, and patient outcomes.

## Methods

### Study Design and Patients

This was a retrospective cohort study, performed at the LUMC, the Netherlands. Patients aged 18 years or older who underwent an MRDTI scan as part of clinical practice for the diagnostic management of suspected thrombosis were included. MRDTI scans performed from its implementation in 2015 up to 30 September 2023 were evaluated in the current study. The Institutional Review Board of the LUMC approved the study.

Relevant patients were identified based on administrative codes, and all patients gave informed consent for the use of their data. The data obtained from the electronic health records included demographic variables and data regarding clinical background, findings of physical examination, results of laboratory tests if performed, radiology reports, MRDTI scan images, and information regarding treatment decisions. Moreover, data were collected on the occurrence of suspected (recurrent) thrombosis during a period of 3 months after the MRDTI scan.

### Objectives

The objective of this study was to evaluate the application of MRDTI in clinical practice, based on our practice at the LUMC. We aimed to assess (1) the frequency of MRDTI scans performed, (2) the indications to perform MRDTI scans, (3) the results of MRDTI scans, (4) how MRDTI scans guided clinical decision-making, and (5) the 3-month incidence of (recurrent) symptomatic thrombosis in patients with MRDTI without signs of acute thrombosis. The main treatment decisions included the decision to start, continue, modify, or discontinue anticoagulant therapy, or to not treat with anticoagulants.

MRDTI scans were performed using a Philips 1.5 Tesla MRI scanner. The MRDTI scan protocols for the lower extremities up to the pelvis and for the upper extremities were evaluated previously in the Theia study and Selene study, in which MRDTI was investigated for the diagnosis of acute recurrent ipsilateral DVT of the leg and diagnosis of upper extremity DVT. 6 9 The technique has an acquisition time of approximately 10 minutes. 6

Acute (recurrent) thrombosis as diagnosed by MRDTI was defined as high signal intensity in the location of a deep vein segment against the suppressed background greater than that observed in the corresponding or contiguous segments of the ipsilateral vein 6 11 12 or high signal intensity in the location of an artery in the case of arterial thrombosis. An MRDTI was considered to rule out acute thrombosis or to indicate the absence of acute thrombosis but rather chronic thrombosis when no high signal intensity in the location of a deep vein or artery was observed.

### Statistical Analysis

Demographic characteristics were described using descriptive statistics. Continuous variables are reported as means with standard deviation or medians with interquartile range, according to their distribution; categorical variables are expressed as frequencies with percentages.

Descriptive analysis was performed to evaluate the frequency of MRDTI scans performed, indications for which the scans were made, the results of the MRDTI scans, and the 3-month incidence of (recurrent) symptomatic thrombosis in patients with an MRDTI showing no signs of acute thrombosis. The frequency of performed MRDTI scans was assessed per year. To evaluate the influence of the MRDTI scans on clinical decision-making, categories of treatment decisions were made and described. All analyses were performed using SPSS version 29.

## Results

### Patients

A total of 36 patients had undergone an MRDTI scan for the diagnostic management of suspected thrombosis between October 2015 and September 2023. The baseline characteristics of these patients are described in Table 1 . The mean age of the study patients was 53 years, and 61% were women. Seven (19%) patients underwent an MRDTI scan while not experiencing symptoms. The other 29 patients presented after a median symptom duration of 3 days.

**Table 1:** Baseline characteristics of 36 patients who underwent an MRDTI scan for diagnostic management of suspected thrombosis

Characteristics	Data ( *n* = 36)
Mean age (± SD), years	53 (18)
Female, *n* (%)	22 (61)
Median duration of complaints (IQR), days	3 (2–12)
MRDTI performed in nonsymptomatic patients, *n* (%)	7 (19)
Prior venous thromboembolism, *n* (%)	27 (75)
One event	17
Two events	5
Three events	2
Four events	2
Five events	1
Type of prior venous thromboembolic events a , *n*	
Deep vein thrombosis of the leg	36
Pulmonary embolism	9
Upper extremity deep vein thrombosis	1
Cerebral vein thrombosis	2
Median time since the last VTE episode (IQR), months	13 (5–50)
Median time since the last DVT episode (IQR), months	25 (5–65)
Active malignancy b , *n* (%)	8 (22)
Immobility >3 d in the past 4 wk, *n* (%)	2 (5.6)
Recent long travel >6 h in the past 4 wk, *n* (%)	0
Trauma or surgery during the past 4 wk, *n* (%)	0
Pregnant, *n* (%)	6 (17)
Known genetic thrombophilia, *n* (%)	4 (11)
Hormone therapy c , *n* (%)	4 (11)
Use of anticoagulant at baseline, *n* (%)	16 (44)
Direct oral anticoagulant	9
Vitamin K antagonist	4
Low molecular weight heparin	3
Use of antiplatelet therapy at baseline, *n* (%)	6 (17)

Abbreviations: DVT, deep vein thrombosis; IQR, interquartile range; MRDTI, magnetic resonance direct thrombus imaging; *n* , number; SD, standard deviation; VTE, venous thromboembolism.

a Total number of prior venous thromboembolic events that patients had experienced; not mutually exclusive.

b Defined as: malignancy diagnosed or treated in the past six months.

c Including estrogenic oral contraceptives and hormonal therapy of malignancy.

### Frequency

The first MRDTI scan in the setting of clinical practice was performed in October 2015, followed by two in 2017 and one in 2018. Since the second half of 2019 after the completion and presentation of the results of the Theia study, MRDTI scanning began to be routinely performed in clinical practice for selected indications, resulting in an increased frequency of MRDTI scans performed per year since 2019 ( Table 2 ). In 2019 and 2020, five MRDTI scans were performed. In 2021 and 2022, eight MRDTI scans per year were performed and in 2023 (up to September 2023), six patients underwent an MRDTI scan. In the same period, 58 MRDTI scans were performed in the setting of research. For reference, during the year 2022, a total of 40 patients were diagnosed with DVT of the leg by CUS in our center, of whom 9 patients had recurrent ipsilateral DVT. One-third (33%) of the patients underwent the MRDTI scan on the same day the MRDTI was ordered, and in 36%, the scan was performed the next day.

**Table 2:** Frequency of MRDTI scans performed between 2015 and 2023 (up to September 2023) and results of the scans, shown per year

Year	Frequency per year, *n*	MRDTI indicating acute thrombosis, per year, *n* (%)
2015	1	1 (100)
2016	0	0 (0)
2017	2	0 (0)
2018	1	0 (0)
2019	5	1 (20)
2020	5	2 (40)
2021	8	4 (50)
2022	8	2 (25)
2023 a	6	3 (50)

Abbreviations: MRDTI, magnetic resonance direct thrombus imaging; *n* , number.

a Up to September 30th, 2023.

### Indications

In the majority of patients, MRDTI scans were performed to differentiate between acute and chronic thrombosis (69%; 25/36). Of these patients, 18 had presented with clinically suspected recurrent ipsilateral DVT and had an inconclusive CUS (i.e., nondiagnostic for the diagnosis of recurrent ipsilateral DVT), which was followed by MRDTI. In two patients, the indication followed from a reference ultrasonography examination performed after the treatment of an initial DVT, which showed signs of acute DVT. This was followed by MRDTI to differentiate between acute recurrent ipsilateral DVT and residual venous abnormalities. Four patients with an incidental finding of thrombosis on an imaging test (ultrasound or computed tomography [CT]; DVT of the leg, iliac vein thrombosis, upper extremity DVT, and thrombosis of the abdominal aorta, respectively) underwent MRDTI to distinguish between acute and chronic thrombi. One patient who experienced recurring DVT (three episodes of DVT of the right leg; one DVT of the left leg) was subjected to MRDTI to assess the age of the residual thrombosis to establish a baseline situation, to anticipate the scenario of another suspected recurrence and a nondiagnostic CUS.

In 10 patients, MRDTI scans were performed to confirm or rule out symptomatic acute DVT, where other imaging tests had not been conclusive on the presence or absence of thrombosis (both CUS and CT scan in one patient; CUS in the other patients). One of these 10 patients had suspected upper extremity DVT that could not be confirmed by ultrasound, and four patients had a clinically suspected first-leg DVT during pregnancy. The remaining five nonpregnant patients were suspected of lower extremity or iliac DVT.

Finally, MRDTI was performed as a primary and single diagnostic imaging test in one patient with suspected recurrent ipsilateral upper extremity DVT.

### MRDTI Scan Results and Treatment Decisions

**Fig. 1:**
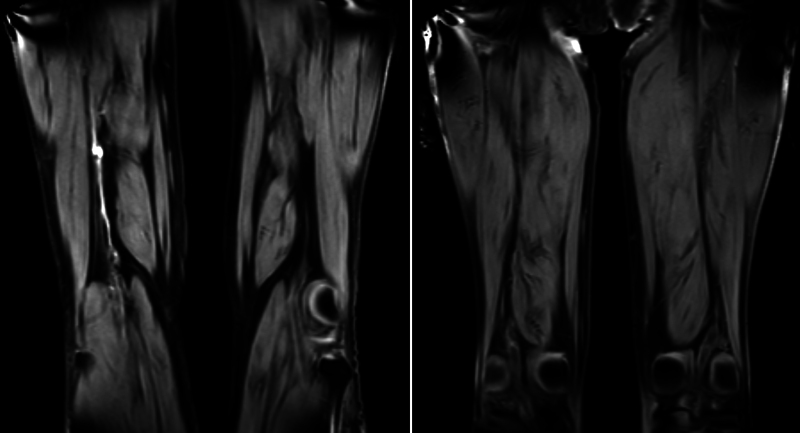
An MRDTI scan indicating acute thrombosis and an MRDTI scan without signs of acute thrombosis. Left: MRDTI scan demonstrating acute thrombosis. A 70-year-old male patient who presented with suspected recurrent ipsilateral DVT of the right leg. CUS revealed thrombosis of the popliteal vein, calf veins, and to the extent assessable, of the superficial femoral vein. MRDTI scan showed asymmetric high signal intensity in the right proximal superficial femoral vein which could be followed up to the popliteal vein and calf veins, indicating acute thrombosis. Right: MRDTI scan demonstrating the absence of acute thrombosis. A 68-year-old male patient who presented with suspected recurrent ipsilateral DVT of the right leg. CUS demonstrated signs of DVT of the right popliteal vein. MRDTI scan showed symmetric low signal intensity along the deep venous system including the femoral vein and popliteal vein of both legs, indicating the absence of acute thrombosis. Abbreviations: MRDTI: magnetic resonance direct thrombus imaging, DVT: deep vein thrombosis, CUS: compression ultrasound.

Acute thrombosis was confirmed by MRDTI in 13 of the 36 patients (36%): recurrent ipsilateral DVT in 9 patients, a first DVT in 3 patients (one had an iliofemoral DVT and two pregnant women had isolated iliac DVT), and a recurrent contralateral DVT in 1 patient which was an incidental finding on an ultrasound examination of both legs performed for another indication. In the other 23 patients (64%), the MRDTI test result indicated the absence of acute thrombosis. The number of positive test results, indicating acute thrombosis, per year is provided in Table 2 . Fig. 1 shows an MRDTI scan demonstrating acute thrombosis and an MRDTI scan without signs of acute thrombosis.

Of the 13 patients with an MRDTI indicating acute thrombosis, 9 started anticoagulant therapy, and anticoagulant treatment was modified in the other 4 patients: 2 switched from direct oral anticoagulant to low molecular weight heparin (LMWH) because of break-through thrombosis in the setting of cancer, 1 patient switched from apixaban to a vitamin K antagonist (VKA), and 1 patient switched from reduced-dose rivaroxaban (10 mg once daily) to VKA.

Of the 23 patients with MRDTI without signs of acute thrombosis, 10 continued the anticoagulants they had used before presentation unchanged. Anticoagulants started shortly before the MRDTI to bridge the time to a final diagnosis were continued in two patients despite the MRDTI scan ruling out acute thrombosis. In one of these two patients, the MRDTI was performed after 2.5 months and an already normalized MRDTI signal could not be ruled out. The other patient was treated with therapeutic anticoagulation for a limited time period because of thrombophlebitis (confirmed on the MRDTI scan). Moreover, one patient, known with triple positive antiphospholipid syndrome, was diagnosed with a thrombophlebitis based on CUS and received LMWH in half-therapeutic dose on top of a VKA for a period of 6 weeks. The CUS revealed a potential recurrent ipsilateral DVT as well, but the MRDTI result, ruling out acute thrombosis, prevented further treatment escalation. The other 10 out of 23 patients did not receive any anticoagulant treatment after the MRDTI result: anticoagulants started to bridge the time to a final diagnosis were discontinued in four patients, one patient had suspected recurrent DVT at the time of finishing treatment of a prior DVT and stopped anticoagulant treatment after the MRDTI without signs of acute thrombosis, and in five patients, who did not use anticoagulants before presentation, no anticoagulant treatment was started.

The MRDTI result thus determined the treatment decision in all, except in two patients who received therapeutic anticoagulation despite MRDTI result excluding acute DVT (34/36; 94%).

### Three-Month Outcomes

One patient had a DVT of the leg within the first 3 months after an MRDTI scan that excluded suspected first upper extremity DVT (42 days following MRDTI). This patient had experienced a prior DVT of the leg 33 years ago and underwent an MRDTI scan after the incidental finding of thrombosis in the left jugular vein on an ultrasound performed for an indication other than thrombosis. The MRDTI did not show signs of upper extremity DVT and jugular vein thrombosis, and no anticoagulation was started. Thus, the 3-month incidence of (recurrent) symptomatic thrombosis in patients with an MRDTI without signs of acute thrombosis was 4.3% (1/23; 95% confidence interval 0.77–21; of note, in four patients with an MRDTI showing no acute thrombosis, data regarding the first 3 months after MRDTI scan were missing).

## Discussion

In this descriptive study, we evaluated the application of MRDTI in routine practice at our hospital, among 36 patients in the routine clinical setting. After the first few MRDTI scans performed between 2015 and 2018 and the completion of the Theia study (published in April 2020), 6 MRDTI has been regularly used in routine practice since the second half of 2019 with a frequency of five to eight scans per year. The most common indication for MRDTI was the differentiation between acute and chronic thrombosis (69%), mainly suspected recurrent ipsilateral DVT of the leg when CUS was inconclusive. In over a third of patients (36%), MRDTI indicated acute thrombosis. The MRDTI result determined the treatment decision in all, except in two patients (94%). Anticoagulant treatment was discontinued, not started or not escalated in patients with an MRDTI result ruling out acute thrombosis, and anticoagulant therapy was started or modified when patients were diagnosed with acute thrombosis based on the MRDTI result.

The diagnostic accuracy of MRDTI has been studied in clinical studies for different venous thromboembolism (VTE) sites. In a prospective study, MRDTI scanning of 101 patients with suspected lower-limb DVT, who were subjected to venography which served as the reference standard, resulted in a sensitivity of 94 to 96% and a specificity of 90 to 92% for DVT. 11 Moreover, interobserver reliability was good (k-statistic 0.89-0.98) and MRDTI scanning was well tolerated. Another prospective study evaluated MRDTI in patients with symptomatic recurrent ipsilateral DVT and asymptomatic patients with chronic residual thrombi of at least 6 months old and demonstrated that the technique could distinguish acute recurrent DVT from chronic thrombi with a sensitivity of 95%, a specificity of 100%, and an excellent interobserver agreement ( *k* = 0.98). 12 Among 30 prospectively included patients with confirmed upper extremity DVT, MRDTI had a sensitivity of 93% and specificity of 100% and was shown a reproducible diagnostic test too (interobserver agreement: *k* = 0.83). 9

Patient outcomes were evaluated in the Theia study, in which patients with suspected recurrent ipsilateral DVT were managed according to the MRDTI result and followed for a period of 3 months. The primary outcome of the Theia study was the 3-month incidence of symptomatic VTE after MRDTI ruling out DVT, which occurred in 1.1% (2/189) among all patients without DVT based on MRDTI and in 1.7% (2/119) among patients with MRDTI showing no signs of DVT and thrombophlebitis who did not receive anticoagulant treatment during the follow-up period. 6 Based on the low incidence of VTE recurrence after MRDTI without signs of acute thrombosis, it was suggested to consider MRDTI for the diagnostic management of suspected recurrent ipsilateral DVT in patients with an inconclusive CUS result. Moreover, based on the reports of reference CUS examinations in Theia study patients with an MRDTI excluding DVT stating that recurrence was likely or could not be excluded, MRDTI could have prevented anticoagulant treatment in 19% of the study population. In the current study, MRDTI scans were evaluated that were performed when the diagnosis of thrombosis, and thus the indication for anticoagulation, was uncertain: we found that 10 patients did not receive any anticoagulant treatment and 10 patients continued the anticoagulants they had used before presentation unchanged after MRDTI ruling out thrombosis. In our study, one patient was diagnosed with DVT of the leg within 3 months after MRDTI showing no signs of upper extremity DVT, but this could be considered a true negative result.

We described one patient who had undergone an MRDTI scan as an additional imaging test to differentiate between acute and chronic thrombosis of the abdominal aorta, which was identified by CT angiography, to establish a treatment plan considering the patient's high bleeding risk. In this case, MRDTI contributed to the treatment decision to not initiate anticoagulant therapy and to continue antiplatelet therapy, as low signal intensity of the aortic thrombus indicating chronic thrombosis was observed. No thrombotic or bleeding complications occurred in the first year after presentation. The case of this patient was described in a previously published case report. 13 Some previous studies evaluated MRDTI for the investigation of carotid atherosclerotic disease and the identification of complicated carotid or upper thoracic aorta plaques (one study demonstrated good interobserver (k-statistic 0.75) and intraobserver (k-statistic 0.9) agreement) 14 15 16 and for the detection of intracoronary thrombi in patients with acute myocardial infarction. 17 A preliminary study investigated MRDTI for acute peripheral arterial occlusion in patients with acute limb ischemia. 4 However, MRDTI has not been investigated for guiding therapeutic management in the setting of arterial thrombosis.

To date, the application of MRDTI is not consistently included in international guidelines. The American College of Chest Physicians 2012 (9th edition) guidelines suggested MRDTI as one of the alternatives to venography in patients with suspected lower extremity DVT when ultrasound is impractical or nondiagnostic, but also mentioned that outcomes of treatment decisions based on MRDTI results were unclear since—at that time—no management studies had been conducted. 18 The American Society of Hematology 2018 guidelines stated research needs for recommendations regarding diagnosis of lower extremity DVT, which included the evaluation of the MRDTI technique to assess acute versus chronic thrombosis. 19

To better determine the potential role of MRDTI in routine practice, the cost-effectiveness of MRDTI was evaluated in a decision analytic model as a predefined secondary analysis of the Theia study, the results of which were published in 2021. 20 This model-based cost-effectiveness analysis showed that diagnostic strategies for suspected recurrent ipsilateral DVT of the leg that included MRDTI scanning resulted in lower 1-year health care costs during the first year of treatment and follow-up compared to strategies without MRDTI scanning, and demonstrated that MRDTI would not lead to higher costs compared with performing ultrasonography only. These findings could inform guideline developers and may contribute to the incorporation of MRDTI in guidelines on the diagnostic management of suspected recurrent ipsilateral DVT.

Our study has limitations. Due to the retrospective design, data regarding the first 3 months after the MRDTI scan were missing in four patients with an MRDTI showing no signs of acute thrombosis. Second, assessing patients' and physicians' satisfaction with the treatment and care they received and provided, respectively, would have been valuable. Such data were not available.

In conclusion, MRDTI has been increasingly used on a regular basis in our daily practice, and its results guided treatment decisions. Suspected recurrent ipsilateral DVT with an inconclusive CUS was the most common indication for MRDTI. MRDTI test results determined treatment decisions in 94% of the patients, and over a third of patients had acute thrombosis confirmed by MRDTI. The findings of this study confirm the clinical impact and feasibility of the application of the MRDTI technique in routine practice.
